# Entire Excision of the Tongue

**Published:** 1891-10

**Authors:** 


### Selections and Abstracts

### ENTIRE EXCISION OF THE TONGUE.

Walter Whitehead (according to *Med. and Surg. Rep.*, Aug. 8th,) reports (*British Med. Journal,*) 104 cases of complete removal of the tongue for carcinoma, with a mortality from the operation of 19.21 per cent. Ages of patients ran from 56 to 70 years. No selection of cases was made for report. He describes the technique as follows:

1. The patient should be completely under the influence of the anæsthetic during the first stage of the operation, but afterwards only partial insensibility should be maintained.

2. The mouth should be securely gagged and kept fully open throughout the operation.

3. The head should be supported in such a position that, while the best light is secured, the blood tends to gravitate out of the mouth instead of backward into the pharynx.

4. A firm ligature should be presssed through the tip of the tongue for the purpose of traction.

5. The first step in the operation consists in dividing the reflection of the mucous membrane between the tongue and jaw and anterior pillars of the fauces.

6. Rapid separation of the anterior portion of the tongue from the floor of the mouth.

7. Securing, if possible, the lingual arteries, with Spencer Wells' forceps, prior to division.

8. Passing the ligature through the glosso-epiglottidean fold before finally separating the tongue.

9. The application of a mercurial solution to the floor of the mouth, followed by painting the surface with an "iodoform stypic varnish."

He recommends early operation, chloroform for an anæsthetic, and the mouth to be kept very widely open. Have the patient sit up from the first, and to walk about should the weather be fine. Scissors are used in preference to other ininstruments.

The ultimate results have always been unsatisfactory, but the author believes that the operation frequently prolongs life, or, failing in this, renders the patient's last days more comfortable. One of the patients survived the operation fourteen years, although he was sixty-two when operated upon.

